# Contribution of *toll*-*like receptor 9* gene single-nucleotide polymorphism to systemic lupus erythematosus

**DOI:** 10.1007/s00296-012-2509-y

**Published:** 2012-09-05

**Authors:** Piotr Piotrowski, Margarita Lianeri, Mariusz Wudarski, Marzena Olesińska, Paweł P. Jagodziński

**Affiliations:** 1 Department of Biochemistry and Molecular Biology, Poznań University of Medical Sciences, 6 Święcickiego St., 60-781 Poznan, Poland; 2 Institute of Rheumatology, Warsaw, Poland

**Keywords:** TLR9, Polymorphisms, SLE

## Abstract

There are several studies on the association of *TLR9* polymorphisms with systemic lupus erythematosus (SLE) in different ethnicities; however, the results are inconsistent. Therefore, we studied the distribution of the *TLR9 C* > *T* (rs352140) polymorphism in patients with SLE (*n* = 254) and controls (*n* = 521) in a Polish population. We did not observe significant differences in the prevalence of the *TLR9 C* > *T* genotype and alleles between patients with SLE and controls. However, we found a contribution of the T/T and T/C genotypes to renal [OR = 2.949 (95 % CI = 1.523–5.711, *p* = 0.001), (*p*_corr_ = 0.017)] and immunologic disorders [OR = 2.938 (95 % CI 1.500–5.755, *p* = 0.0012), (*p*_corr_ = 0.0204)] in SLE patients. Moreover, we observed a significant association between the *TLR9* T/T and T/C genotypes and the presence of anti-dsDNA Ab [OR = 3.682 (1.647–8.230, *p* = 0.001), (*p*_corr_ = 0.017)]. Our studies suggest that the *TLR9 C* > *T* (rs352140) polymorphism might contribute to renal and immunologic disorders and to the presence of anti-dsDNA Ab.

## Introduction

Systemic lupus erythematosus (SLE) is a progressive and chronic autoimmune disorder in which the immune system cannot distinguish between the body’s own tissues and foreign antigens, producing antibodies (Ab) to self-antigens [1]. Flare-ups of SLE can be initiated by disparate environmental factors, such as exposure to ultraviolet light, drugs, chemicals, as well as viral and bacterial infections [2, 3]. The underlying cause of SLE is elusive; however, it is well established that both environmental and genetic factors are involved in this disease [3–7]. Genome-wide association studies have revealed many SLE prone genes and the contribution of some of these genes to the risk of SLE have been studied in different ethnicities [7]. One of these genes is the *toll-like* *receptor 9* (*TLR9*) gene located in susceptibility regions for SLE [7].

TLR9 plays an elementary role in pathogen recognition and activation of innate immunity [8–10]. This receptor recognizes unmethylated cytosine–phosphate–guanine (CpG) dinucleotide motifs located in bacterial, viral and fungal DNA [8–12]. The *TLR9* gene is expressed in macrophages and dendritic cells, and TLR9 has been shown to be present almost exclusively in endosomes [8, 13–15].

It has been demonstrated that TLR9 is involved in the development of autoimmunity in SLE patients [16–18]. Stimulation of TLR9 in the endosomes by host DNA leads to plasmacytoid dendritic cell activation and type I interferon biosynthesis, which is implicated in lupus pathophysiology [19]. TLR9 activation also promotes the production of IgG2a and IgG2b autoantibodies recognizing host DNA, which further develop autoimmunity pathology [20]. The role of TLR9 in autoimmunity was also demonstrated by Leadbetter et al. [21], who indicated that DNA-containing complexes interacting with TLR9 may activate both autoreactive B cells and other antigen presenting cells.

There are several studies on the contribution of *TLR9* polymorphisms to the risk of SLE in different ethnicities; however, the results are inconsistent [22–27]. The four *TLR9* single-nucleotide polymorphisms rs187084, rs5743836, rs352139 and rs352140 in Caucasians are located in the same block of linkage disequilibrium (LD) HapMap CEU data (http://hapmap.ncbi.nlm.nih.gov/). Therefore, we aimed to study whether *TLR9* C > T (rs352140) can be a genetic risk factor of SLE in the Polish population. Because SLE is a heterogeneous disorder, we also evaluated the contribution of this polymorphism to different clinical symptoms of SLE.

## Patients and methods

### Patients and controls

Data for two hundred and fifty-four women fulfilling the American College of Rheumatology Classification criteria for SLE [28, 29] were collected in a random manner for the study at the Institute of Rheumatology in Warsaw, Poland. Controls included five hundred and twenty-one unrelated healthy volunteers and healthy women selected during medical examination at the Institute of Mother and Child, Warsaw. Women with SLE and controls were of Polish Caucasian origin and of a similar age. The mean age of SLE patients at diagnosis was 36 ± 9 years and of controls 35 ± 8 years. All participating subjects provided written consent. The study procedures were approved by the Local Ethical Committee of Poznań University of Medical Sciences.

### Genotyping

DNA was isolated from peripheral leucocytes using a standard salting out procedure. Identification of the *TLR9* C > T (rs352140) polymorphic variant was performed by polymerase chain reaction–restriction fragment length polymorphism (PCR–RFLP). PCR was conducted employing primer pair 5′ GCAGCACCCTCAACTTCACC 3′and 5′ GGCTGTGGATGTTGTTGTGG 3′. The PCR-amplified fragments of *TLR9* that were 360 bp in length were isolated and digested with the endonuclease BstUI (CG/CG) New England BioLabs (Ipswich, USA). The *TLR9* C allele was cleaved into 227 bp and 133 bp fragments, whereas the *TLR9* T allele remained uncut. DNA fragments were separated by electrophoresis on 3 % agarose gel and visualized by ethidium bromide staining. The *TLR9* C > T polymorphism was confirmed by repeated PCR–RFLP. Moreover, the restriction analysis was confirmed by commercial sequencing analysis.

### Statistical analysis

The distribution of genotypes in patients and controls was examined for deviation from Hardy–Weinberg equilibrium using exact and log likelihood ratio Chi-squared (χ^2^) tests (http://ihg.gsf.de/cgi-bin/hw/hwa1.pl). The polymorphism was tested for association with SLE incidence using the χ^2^ test for trend (*p*_trend_). The χ^2^ test was employed to examine differences in genotypic and allelic distribution between patients and controls, and a *p* value <0.05 was considered statistically significant. The odds ratio (OR) and 95 % confidence intervals (95 % CI) were calculated. Contribution of the *TLR9* C > T polymorphism to clinical manifestations and the production of autoantibodies were determined by χ^2^ test. The Bonferroni correction for multiple comparisons was used and both *p* values, before (*p*) and after correction (*p*_corr_), were determined.

## Results

### Distribution of the *TLR9* C > T (rs352140) polymorphism in SLE patients and healthy individuals

Prevalence of the *TLR9* C > T genotypes did not exhibit significant deviation from Hardy–Weinberg equilibrium between patients and controls. We observed a similar frequency of the *TLR9* T/T genotype in patients with SLE and healthy individuals, and OR for SLE patients with the T/T versus T/C and C/C genotypes was 1.079 (95 % CI = 0.7592–1.534, *p* = 0.6713) (Table 1). The frequency of the *TLR9* T/T and C/T genotypes was not statistically increased in patients with SLE than in controls OR = 1.414 (95 % CI = 0.9847–2.029, *p* = 0.0598) (Table 1). We also observed a non-significant increase in the *TLR9* T allele in patients compared to healthy individuals OR = 1.164 (95 % CI = 0.9412–1.439, *p* = 0.1610) (Table 1). The *p* value of the χ^2^ test of the trend observed for the *TLR9 C* > *T* polymorphism was also not significant (*p*_trend_ = 0.1531) (Table 1).

**Table 1:** Prevalence of the *TLR9 C* > *T (rs352140)* polymorphism in SLE patients and controls

*TLR9 C* > *T* (*rs352140*)	SLE *n* = 254	Controls *n* = 521	OR	95 %CI	*p* value^d^	*p*_trend_
Genotype frequency						0.1531
C/C	52 (0.21)	139 (0.27)				
C/T	140 (0.55)	262 (0.50)				
T/T	62 (0.24)	120 (0.23)	1.079^a^	(0.7592–1.534)^a^	0.6713^a^	
C/T + T/T	202 (0.79)	382 (0.73)	1.414^b^	(0.9847–2.029)^b^	0.0598^b^	
Minor allele frequency						
T	0.52	0.48	1.164^c^	0.9412–1.439^c^	0.1610^c^	

The odds ratio (OR) was calculated for patients

^a ^(T/T vs C/T and C/C genotype), ^b ^(T/T and C/T vs C/C genotype). We also determined the OR for the patients’ minor allele; ^c ^(T allele vs C allele); ^d ^χ^2^ test

Association of the *TLR9* C > T (rs352140) polymorphism with clinical manifestations and production of autoantibodies in patients with SLE.

We observed a contribution of the T/T and T/C genotypes to renal OR = 2.949 (95 % CI = 1.523–5.711, *p* = 0.001), (*p*_corr_ = 0.017) and immunologic disorders OR = 2.938 (95 % CI 1.500–5.755, *p* = 0.0012), (*p*_corr_ = 0.0204) in SLE patients (Table 2). Moreover, we observed a significant association between the *TLR9* T/T and T/C genotypes and the presence of anti-dsDNA Ab OR = 3.682 (1.647–8.230, *p* = 0.001), (*p*_corr_ = 0.017) (Table 3).

**Table 2:** Distribution of the *TLR9* C > T (rs352140) polymorphism among SLE patients with different clinical manifestations

Characteristic	Genotype distribution			Odds ratio (95 % CI), *p* ^c^
	C/C (52)^a^	C/T (140)^a^	T/T (62)^a^	
Malar rash	29	77	33	
Discoid rash	16	43	18	
Phototosensitivity	26	64	28	
Oral or nasopharyngeal	23	53	26	
Arthritis	14	33	15	
Serositis	10	25	11	
Renal	15	76	34	2.949 (1.523–5.711, *p* = 0.001)^b^
Neurologic	12	27	13	
Hematologic	19	47	21	
Immunologic^d^	14	76	29	2.938 (1.500–5.755, *p* = 0.0012)^b^
ANA	52	140	62	

^a^Represents the absolute number of positive patients for C**/**C, C**/**T and T**/**T genotypes, respectively. Comparison of genotypes ^b ^(T**/**T or C**/**T vs C**/**C genotype) between patients with and patients without a particular manifestation was performed by ^c ^χ^2^ test. ^d ^Include presence of anti-DNA Ab or anti-Smith Ab or antiphospholipid Ab

**Table 3:** Effect of the *TLR9* C > T (rs352140) polymorphism on the presence of various autoantibodies in patients with SLE

Autoantibodies	Genotype distribution			Odds ratio (95 % CI), *p* ^c^
	C/C (52)^a^	T/C (140)^a^	T/T (62)^a^	
Anti-dsDNA	8	58	23	3.682 (1.647–8.230, *p* = 0.001)^b^
Anti-Smith	4	13	6	
Anti-snRNP	10	28	13	
Anti-Ro	9	24	11	
Anti-La	8	20	8	
Anti-Scl-70	10	27	12	

^a^Represents the absolute number of positive patients for C/C, C/T and T/T. Genotype comparison ^b ^(T/T and T/C vs C/C genotype) between patients with and patients without an autoantibody was performed by ^c ^χ ^2^ test

## Discussion

Some studies have demonstrated increased *TLR9* expression in B cells from patients with SLE [30, 31]. Papadimitraki et al. [30] reported an increased proportion of peripheral blood memory B cells and plasma cells expressing TLR9, which correlated with the presence of anti-dsDNA Ab in patients with active SLE. Another study revealed that the level of TLR9 mRNA in B cells was increased in SLE patients, and TLR9 expression on CD20^+^ B cells correlated with SLE activity and CH50 [31]. This may suggest that genetic variations of *TLR9* that affect their expression may have an effect on SLE development and clinical manifestation of this disease.

We did not observe a contribution of the *TLR9* C > T (rs352140) polymorphism to the risk of SLE in a Polish population. Recent studies conducted by Huang et al. (2011) suggested that the TLR9 −1486 T/C (rs187084) polymorphism, located in the LD block with rs352140, is related to SLE in Taiwanese patients. [22]. In addition to this finding, Xu et al. [23] demonstrated a significant association of rs352140 gene variants with the susceptibility to SLE in a Chinese population. Additionally, the TLR9 G allele at position +1174 of TLR9 (rs352139) conferred an increased risk for SLE in a Japanese population [24]. The contributions of rs5743836 to SLE have been observed in individuals of European descent from Southern Brazil; however, this was not confirmed in Caucasian American individuals [25, 32]. *TLR9* polymorphisms were not significantly associated with the susceptibility to SLE and related phenotypes in Korean patients with SLE [26]. Furthermore, Zhou et al. [27] did not find a significant contribution of the rs352140 polymorphism to SLE development in a Chinese Han population.

In our study, the *TRL9* T/T and T/C genotypes exhibited a significantly increased risk of developing renal disease in patients with SLE. The significant association between the rs352140 gene variant and lupus nephritis was also observed in a Chinese Han population [27]. We also observed that the *TRL9* T/T and T/C genotypes were significantly associated with immunologic disorders and the presence of anti-dsDNA Ab in patients with SLE. To date, an increased frequency of the *TRL9* rs5743836 C allele has been observed in patients of European descent from Southern Brazil bearing the Anti-SSa/Ro Ab [25].

These differences in the effect of the *TRL9* polymorphisms on SLE development and clinical manifestations in various populations may be due to racial heterogeneity, the size of the studied groups, or population exposure to disparate environmental factors.

The function of +1174 G/A of *TLR9* rs187084 located in the LD block with rs352140 has been studied by Tao et al. [24], who demonstrated that the +1174 G variant may down-regulate *TLR9* expression. Moreover, they indicated that *TLR9* rs187084 may contribute to differences in the B cell response to autoantigens and the production of autoantibodies [24].

Recently, the role of *TLR9* expression in the production of anti-dsDNA Ab was confirmed by Chen et al. [33], who performed knockdown of TLR9 by siRNA in B cells, resulting in a reduction of anti-dsDNA Ab levels and amelioration of the disease in SLE murine model. Recent studies also suggest that the changes in *TLR9* expression may have an effect on renal disease development in SLE [34, 35]. Machida et al. [34] demonstrated that injured podocytes express *TLR9* in active lupus nephrites accompanied by proteinuria and elevated anti-dsDNA Ab. In addition to this finding, Anders et al. [35] revealed that activation of TLR9 by CpG oligonucleotides in MRL-Fas (lpr) mice induces anti-dsDNA Ab production and renal disease.

Our study may suggest that renal disease and immunologic disorders, along with the presence of anti-dsDNA Ab, in SLE patients may be associated with the *TRL9* (rs352140) T gene variant. However, to confirm the role of the rs352140 polymorphism in SLE, this study should be replicated in a larger and independent cohort.
